# Comprehensive insights on how 2,4-dichlorophenoxyacetic acid retards senescence in post-harvest citrus fruits using transcriptomic and proteomic approaches

**DOI:** 10.1093/jxb/ert344

**Published:** 2013-11-08

**Authors:** Qiaoli Ma, Yuduan Ding, Jiwei Chang, Xiaohua Sun, Li Zhang, Qingjiang Wei, Yunjiang Cheng, Lingling Chen, Juan Xu, Xiuxin Deng

**Affiliations:** ^1^Key Laboratory of Horticultural Plant Biology of Ministry of Education, Huazhong Agricultural University, Wuhan 430070, PR China; ^2^Center for Bioinformatics, College of Life Science and Technology, Huazhong Agricultural University, Wuhan 430070, PR China

**Keywords:** ABA, auxin, citrus fruit, 2,4-dichlorophenoxy acetic acid (2,4-D), ethylene, fruit quality preservation, fruit senescence, plant hormones, stress defence.

## Abstract

Auxin-like 2,4-dichlorophenoxyacetic acid (2,4-D), a high-efficiency anti-stalling agent for the post-harvest fresh fruit industry, has had its use restricted due to environmental concerns. However, no other substitutes for 2,4-D are available to the post-harvest industry. Insights into the molecular mechanism underlying the effects of 2,4-D on fruit quality preservation will provide a theoretical basis for exploring new safe and effective anti-stalling agents. This study comprehensively analysed changes in the peel of Olinda Valencia orange [*Citrus sinensis* (L.) Osbeck] induced by 500 ppm 2,4-D using ‘omic’-driven approaches. Transcriptional profiling revealed that transcriptional factor (mainly AP2/ERF, WRKY, and NAC family members), transport, and hormone metabolism genes were over-represented and up-regulated within 24h post-treatment (HPT). Stress defence genes were up-regulated, while cell wall metabolism genes were down-regulated after 48 HPT. However, secondary metabolism genes, especially phenylpropanoid and lignin biosynthesis-related genes, were over-represented at all the time points. Comparative proteomic analysis indicated that the expression of proteins implicated in stress responses (25%), hormone metabolism, and signal transduction (12%) significantly accumulated at the post-transcriptional level. Hormone levels detected by high-performance liquid chromatography–tandem mass spectrometry (HPLC-MS/MS) showed that abscisic acid, salicylic acid, and 2,4-D significantly increased, while ethylene production (detected by gas chromatography) decreased after 2,4-D treatment. In addition, lignin and water content in the fruit peel also increased and the epicuticle wax ultrastructure was modified. In conclusion, 2,4-D retarded fruit senescence by altering the levels of many endogenous hormones and by improving stress defence capabilities by up-regulating defence-related genes and proteins.

## Abstract (precis)

2,4-D retards senescence of postharvest citrus fruits by increasing exogenous auxin, endogenous ABA and SA contents, while decreasing ethylene production; and enhancing stress-defense capability through changing epicuticular wax morphology and lignin content in peel

## Introduction

2,4-Dichlorophenoxyacetic acid (2,4-D) is an auxin-like plant growth regulator. Since the 1940s, 2,4-D and its derivatives have been widely used during citrus post-harvest handling to maintain fruit quality. Commercially, the sodium salt of 2,4-D is usually applied to the post-harvest citrus fruit in a dip treatment at 500 ppm (Cronjé *et al.*, 2004), which effectively retards calyx abscission and represses pathogens growing into the fruit when the button is separated from the fruit (Carvalho *et al.*, 2008). It has been reported that 2,4-D has a broad-spectrum antifungal activity *in vitro* (Erickson *et al.*, 1958) and could effectively reduce black rot incidence in citrus fruits during storage (DeWolfe *et al.*, 1959). It also reduces fruit weight loss, retains rind firmness, and extends storage life (Ferguson *et al.*, 1982). However, 2,4-D is prohibited in the citrus post-harvest industry in many countries because of concerns over its effects on human health and environmental safety. Therefore, understanding the mechanism of molecular regulation behind 2,4-D’s effect on fruit quality preservation may provide information that will improve the search for effective, non-toxic alternatives.

Recently, the precise regulatory mechanism behind natural auxin has been discovered. Auxin response factors (ARFs), together with auxin/indole acetic acid proteins (Aux/IAAs), are transcription factors (TFs) that play important roles in regulating auxin-responsive transcription in plants. At low concentrations, ARF activators residing on the promoters of auxin-responsive genes are inactive because they were bound to the Aux/IAA transcription repressors. Exogenous 2,4-D is able to bind specifically to the TIR1 auxin receptors (Kepinski and Leyser, 2005; Parry *et al.*, 2009) and acts as a ‘molecular glue’ that strengthens the interaction between Aux/IAA and the ubiquitin protein complex, SCF^TIR1^. It promotes the degradation of Aux/IAA and then allows the ARF activators to reactivate/activate the auxin response genes (Tan *et al.*, 2007).

More recently, 2,4-D-induced genes and proteins downstream of the receptors have been described in detail. Raghavan *et al.* (2005, 2006) comprehensively surveyed the regulation of the entire *Arabidopsis* genome in response to a range of 2,4-D concentrations using microarray and found that the genes involved in auxin, abscisic acid (ABA), and ethylene biosynthesis and signalling (especially TFs) had been significantly altered. Teixeira *et al.* (2005) and Fanous *et al.* (2007) used two-dimensional polyacrylamide gel electrophoresis (2-D PAGE) to survey 2,4-D-responsive proteins in microorganisms and found that some proteins involved in stress responses and intracellular pH homeostasis were induced, including heat shock proteins (HSPs) and V-ATPase components.

Exogenous 2,4-D also alters plant hormones levels (Pazmiño *et al.*, 2012). Ethylene biosynthesis and signal transduction were activated as a result of the up-regulation of genes encoding 1-aminocyclopropane-1-carboxylic acid synthase (ACS) and ACC oxidase (ACO) and the down-regulation of the negative regulator, *CTR1* (Raghavan *et al.*, 2006). ABA biosynthesis was then triggered by an ethylene burst causing the overexpression of 9-*cis*-epoxycarotenoid dioxygenase (NCED), which is a key enzyme in the ABA biosynthesis pathway (Kraft *et al.*, 2007; Grossmann, 2010). 12-Oxophytodienoate reductase 2 (OPR2), which is involved in jasmonate biosynthesis, was also induced by 2,4-D (Raghavan *et al.*, 2005). Furthermore, excess 2,4-D induced the overproduction of toxic reactive oxygen species (ROS) and lipid peroxidation, which is associated with an increase in antioxidants and the activation of antioxidative enzymes, such as peroxidase (Karuppanapandian *et al.*, 2011; Pazmiño *et al.*, 2011). Genes encoding stress- and defence-related proteins, such as HSP70 (Raghavan *et al.*, 2005), chitinase, pathogenesis-related protein PrP4A, HSP71.2, and HSP71.1 (Pazmiño *et al.*, 2011), were also induced by 2,4-D. A genome-wide transcriptional analysis of wheat (a 2,4-D-resistant plant) revealed that phenylpropanoid metabolism-related genes were significantly induced by herbicidal concentrations of 2,4-D (Pasquer *et al.*, 2006), particularly genes involved in lignin and flavonoid biosyntheses, such as phenylalanine ammonia lyase (PAL), caffeic acid *O*-methyltransferase (COMT), cinnamate 4-hydroxylase (C4H), chalcone synthase, *S*-adenosylmethionine synthetase, and peroxidase.

In this study, ‘omics’ approaches were used to investigate the effects of 2,4-D on Olinda orange fruit peel at the gene transcription, protein expression, and hormone metabolite levels. The objectives were to gain further insights into the molecular regulation mechanism controlling 2,4-D effects on post-harvest fruit quality preservation and provide new information that will improve the search for new, safer, and efficient alternatives to 2,4-D.

## Materials and methods

### Plant material sampling

Commercially mature fruits of Olinda Valencia orange [*Citrus sinensis* (L.) Osbeck] were randomly harvested from a commercial orchard in Yichang, Hubei Provence, China, in 2009, 2010, and 2012. Only fruits with uniform colour and size, that were free from any visible injury or blemishes, were selected as materials. About 300kg of fruits were divided equally into two groups and were dipped either in water or in 500 ppm 2,4-D aqueous solution for 2min. They were then stored under the same conditions at room temperature and 85–90% relative humidity for >2 months.

At each sampling stage, mixed pericarp (consisting of flavedo and albedo) from 15 fruits in each group was collected at 0, 12, 24, 48, and 72h post-treatment (HPT), immediately frozen in liquid nitrogen, and stored at –80 °C.

### RNA isolation and microarray hybridization

Two total RNA samples were independently isolated from each pericarp sample, according to the method described by Liu *et al.* (2009), and hybridized with commercial genechip^®^ Citrus Genome Arrays (Cat. #900732; Affymetrix^®^; Santa Clara, CA, USA).

### Gene annotation and functional classification

The differentially expressed probe sets were annotated based on sequence similarity by blast (BLASTx, version 2.2.21) to *Arabidopsis* protein release TAIR 9 (e^–10^) (ftp://ftp.arabidopsis.org/home/tair/Genes/TAIR9_genome_release/) using the default settings. A total of 2133 genes were classified into 34 MapMan catalogues (version 3.5.1, http://mapman.gabipd.org/).

### Assessment of over-represented gene categories

To assess the significance of over-represented categories, *P*-values for the numbers of probe sets in the 33 categories at each time point or in total were computed based on their hypergeometric distribution. A *P*-value <0.001 was considered significant.

### Total protein extraction, two-dimensional gel electrophoresis, and image analysis

Three total protein samples were independently extracted from each pericarp sample. The protein extraction, 2-D PAGE, image analysis, and differential spot identification were performed according to Yun *et al.* (2012).

### Lignin content

The lignin in the pericarp was measured at 3 and 30 days post-treatment (DPT) according to the method described by Nafussi *et al.* (2001). Lignin content was the mean of three replicate samples ±SE, and was expressed as absorbance at 280nm g^–1^ of fresh weight tissue.

### Water content

The water content of the pericarp was measured according to the method described by Cajuste and Lafuente (2007). Pericarp disks were cut using a 1.2cm diameter cork borer from the equatorial plane of 18 fruits and were dried in an oven at 100 ºC until they achieved a constant weight. The difference between the fresh weight and dry weight was used to calculate the total water content.

### Pericarp sample preparation for scanning electron microscope observation

Exactly 2mm^2^ square pericarps were excised from the equatorial plane of five fruits, fixed with 2.5% glutaraldehyde for 24h at 4 ºC, washed with phosphate buffer (pH 7.2), dehydrated twice with a series of ethanol solutions (30, 50, 70, 85, 95, and 100%), and dried using a critical point dryer and liquid CO_2_. The dried samples were placed on a metallic support, coated with a thin layer of gold (20–30nm), and observed using a scanning electron microscope JSM-6390LV (JEOL, Japan) operated at 10kV.

### Respiration rate determination

The fruit respiration rate was detected using a fruit and vegetable respiration rate infrared CO_2_ determinator (GXH-3051H, Beijing, China). The result was taken as the mean of three replications ±SE and was expressed in mg CO_2_ kg^–1^ h^–1^.

### Measurement of ABA, SA, and 2,4-D content

ABA, salicylic acid (SA), and 2,4-D in Olinda orange fruit peel were extracted according to Pan *et al.* (2010). A 10ng aliqout of [^2^H_6_]ABA (2-*cis*, 4-*trans*-abscisic acid-[^2^H_6_], olchemim, Czech Republic) and [^2^H_4_]SA (2-hydroxy-benzoic acid-[^2^H_4_], olchemim, Czech Republic) were respectively used as internal standards for ABA and SA detection. Hormone extracts were separated by high-perfomance liquid chromatography (HPLC; Agilent 1100, Agilent Technologies, Palo Alto, CA, USA) and measured with HPLC-electrospray ionization-mass spectrometry (ESI-MS) (API3000 mass spectrometer, Applied Biosystems, Foster City, CA, USA), and the MS/MS condition of each analyte was set according to Pan *et al.* (2010). MS/MS conditions for quantifying 2,4-D were: negative scan mode, Q1: 220.80/Q3: 163.10 (80ms); DP –45V; CE –21V and EP –10V; CXP –11V.

### Ethylene measurement

Ethylene release was determined using an Agilent 7890 series gas chromatograph (Agilent Technologies) with a flame ionization detector (Cheng *et al.*, 2012). Three biological replications of 6–8 fruits were sealed in a 2.5 litre plastic container for 8h. Exactly 1ml of gas sample was injected into the gas chromatorgraph using a syringe, and this was replicated three times for each biological sample. Ethylene production was expressed as μl kg^–1^ h^–1^.

## Results

### Effects of 2,4-D on the incidence of fruit decay and the respiration rate

**Fig. 1.**
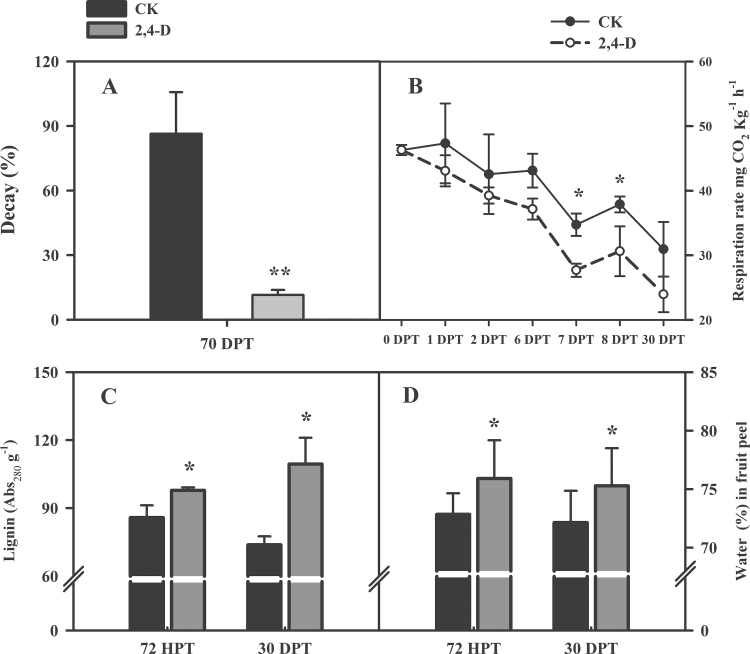
Fruit decay incidence (A) at 70 DPT; fruit respiration rate (B) at 30 DPT; acetyl bromide-soluble lignin (C) and water contents (D) in fruit peels at 72 HPT and 30 DPT.

Fruit decay incidences were significantly reduced by 2,4-D. The rot rate of the control fruit reached 86.37% at 70 DPT, while it was only 11.58% in the treated fruits in 2012 (Fig. 1A). A similar result was also obtained in 2010 (data not shown). Both the control and treated fruits showed a gradual decrease in respiration rate during the storage period, but the respiration rate in 2,4-D-treated fruits was significantly inhibited at 7 and 8 DPT (Fig. 1B), compared with the control.

### Transcriptome characteristics after 2,4-D treatment

**Fig. 2.**
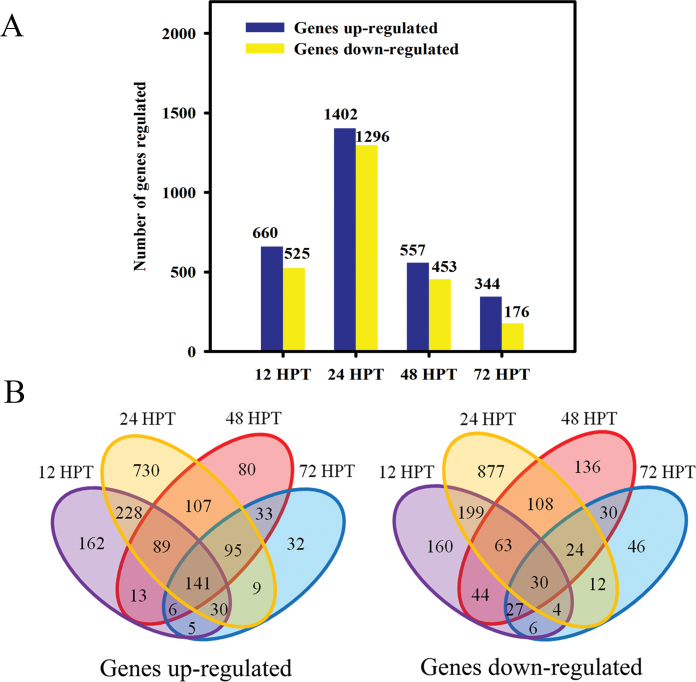
(A) Number of genes differentially expressed after 2,4-D treatment at 12, 24, 48, and 72 HPT. The two adjacent sets of bars represent the up- and down-regulated genes, respectively. (B) Venn diagram showing the overlap/non-overlap of up- or down-regulated genes at 12, 24, 48, and 72 HPT. (This figure is available in colour at *JXB* online.)

The pericarps of Olinda orange fruit exposed to 500 ppm 2,4-D at 12, 24, 48, and 72 HPT were used for transcriptional profile analysis. In total, 3413 probe sets were differentially expressed between the control and the treated samples [*P* < 0.05, and fold change (FC) >2], and 1185, 2698, 1010, and 520 probe sets were differentially expressed at 12, 24, 48, and 72 HPT, respectively (Fig. 2A). The number of differentially expressed genes increased from 12 HPT, peaked at 24 HPT, but sharply decreased from 48 HPT to 72 HPT. Both up- and down-regulated gene numbers showed peaks at 24 HPT, which may result from the time scale required for 2,4-D absorption, accumulation, and biodegradation in cells (Fig. 7A). The total number of up-regulated genes was ~8–95% more than that of down-regulated genes over the four time points.

About 6–14% of the total number of regulated genes were specifically up-regulated at one time point and 141 genes were up-regulated consistently over the 72h (Fig. 2B). About 9–33% of the total number of regulated genes were specifically down-regulated at one time point and 30 genes were consistently down-regulated over the 72h (Fig. 2B).

### Microarray data validation

Quantitative real-time PCR (qRT-PCR) was performed in order to validate the microarray data (Supplementary Fig. S1 available at *JXB* online). The microarray and qRT-PCR expression patterns were similar for most of the 12 genes of interest. This confirmed that the microarray data were reliable.

### Changes in co-expression patterns between the control and the 2,4-D-regulated genes

**Fig. 3.**
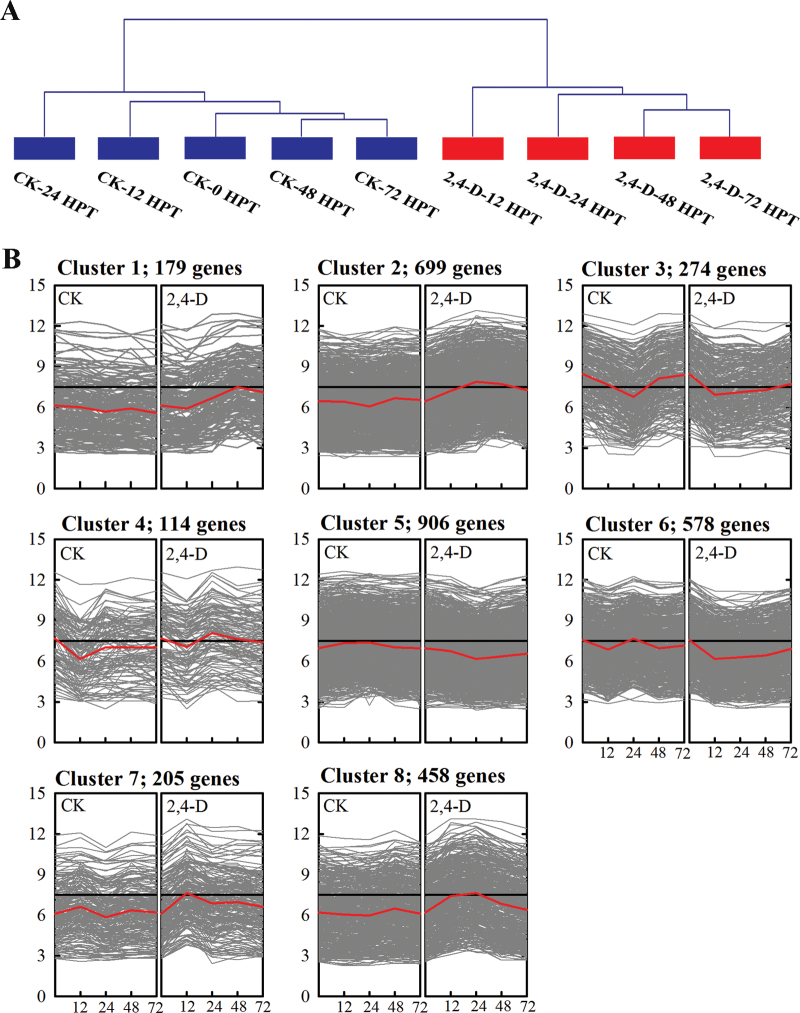
Time-course gene expression patterns of the differentially expressed genes induced by 2,4-D based on hierarchical clustering (A) and K-means clustering (B). (This figure is available in colour at *JXB* online.)

Cluster analyses were performed using TM4:mev4.7.4 software. Hierarchical cluster analysis of 3413 probe sets revealed that the nine samples were distributed in two distinct clusters: a control group and a treatment group (Fig. 3A). This indicated that 2,4-D significantly altered gene expression patterns in fruit peel. K-means clustering was conducted in order to analyse the changes in co-expression patterns between the controls and the treatments, and eight distinct time-course clusters were identified (Fig. 3B; Supplementary Table S1 at *JXB* online). In the control samples, the genes in clusters 1, 2, 5, 6, 7, and 8 showed no changes within 72 HPT and the genes in clusters 3 and 4 showed significant down-regulation at 24 and 12 HPT, respectively. However, in the 2,4-D-treated samples, the genes in clusters 1 and 7 were considerably up-regulated at 48 and 12 HPT, respectively, and the genes in cluster 2 or 8 were up-regulated at both 24 and 48 HPT or 12 and 24 HPT, respectively. The genes in clusters 3 and 6 were consistently down-regulated at 48 HPT.

### Functional category and over-representation of gene ontology terms

**Fig. 4.**
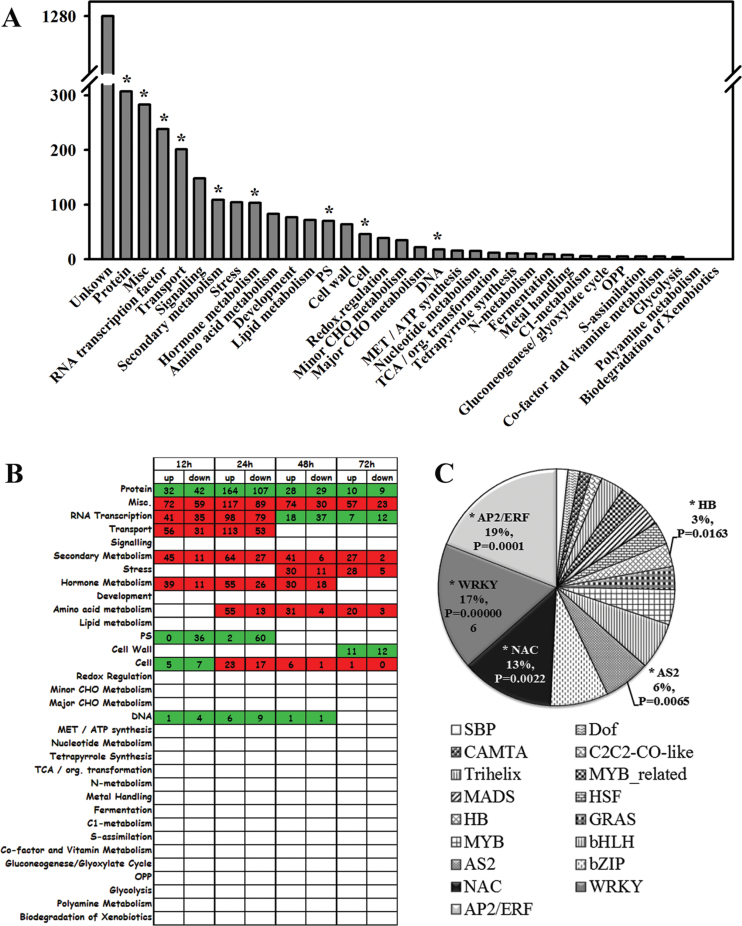
(A) Distributions and functional categories of the 3143 differentially expressed genes based on MapMan. (B) Distributions of the over-represented gene categories at different time points. Numbers in the squares indicate the number of over-represented up- or down-regulated genes in the corresponding functional categories. (C) The pie chart represents the family names and relative proportions of up-regulated transcription factors. * represents the over-represented categories (*P* < 0.001) or the transcription factors (*P* < 0.05) based on the hypergeometric distribution algorithm. (This figure is available in colour at *JXB* online.)

In total, 2133 probe sets were annotated to one or more functional genes and they were classified into 34 MapMan catalogues based on their sequence similarity (Fig. 4A; Supplementary Table S2 at *JXB* online). The nine over-represented (based on a hypergeometric distribution algorithm, *P* < 0.001) catalogues were: protein, Misc, RNA transcription, transport, secondary metabolism, hormone metabolism, photosynthesis, cell, and DNA. These biological processes were primarily affected by the 2,4-D treatment.

In order to understand the biological significance of the genes that were coordinately regulated at different periods, the over-represented categories at each time point are displayed in Fig. 4B. Genes involved in RNA transcription (especially TFs), substance transport, and hormone metabolism were significantly induced and over-represented within 24h. This suggests that these genes may have participated in 2,4-D accumulation and perception and signal transduction, which set up the cascade responses to the earlier gene inductions. Stress-responsive genes and genes related to amino acid and cell metabolism were up-regulated and over-represented at 48 and 72 HPT. The genes related to secondary metabolism, miscellaneous enzyme families and protein synthesis, modification, targeting, and degradation were constantly over-represented over the 72h period. It was inferred that genes differentially expressed at later time periods may be the result of cascade responses initiated by earlier biological processes.

### Effect of 2,4-D on hormone signalling and response-related gene expression

The expression of several genes involved in auxin perception, signalling, response, and homeostasis was altered by 2,4-D treatment (Supplementary Table S3 at *JXB* online). One of the auxin receptor genes, *AFB5* (auxin F-box-binding factor), was up-regulated at 24 HPT. AUX/IAAs are repressors of ARFs which are the TFs of auxin-responsive genes. *ARF4* was not induced, but *ARF6* and *ARF10* were induced at 24 and 48 HPT, respectively, and *IAA4* and *IAA16* were differentially regulated at each time point. *GH3.1*, an early auxin-responsive gene encoding an IAA-amido synthetase that conjugates IAA to amino acids, was highly and persistently up-regulated by >2- to 32-fold within 72 HPT. *IAR3* and *ILL2*, which encode amidohydrolases that cleave IAA conjugates to yield free IAA, and *JAR1*, which encodes an enzyme that conjugates jasmonic acid (JA) to isoleucine, were induced before 24 HPT. Other unknown auxin-responsive genes and *ATB2* were also up-regulated. However, *aldo/keto reductase* and *AGD2* genes were down-regulated.

NCEDs are the key enzymes in the ABA biosynthesis pathway. Type 2C protein phosphatases (PP2Cs) and protein kinase SnRKs are the key regulators involved in ABA signalling. In this study, the negative regulator, putative *PP2C*, was down-regulated at 24 HPT, but *NCED3*, *NCED4* (Cit.8156.1.S1_at), *NCED5*, *SnRK2.6* (positive regulator in ABA signalling), and an ABA-responsive gene encoding GRAM domain-containing protein were significantly up-regulated before 24 HPT (Supplementary Table S3 at *JXB* online). This indicated that ABA biosynthesis and signalling were induced in fruit peel by 2,4-D treatment. Furthermore, genes encoding key enzymes in the ethylene biosynthesis pathway, such as *ACO4*, *ACS1*, *ACS6*, and eight *2OG-Fe(II) oxygenase* family members, were significantly induced. Transcriptional regulators that participate in ethylene perception and signal transduction were also induced, for example *ERF1*, *ERF2*, *ERF12*, *ERS1* (ethylene receptor), and putative *AP2 domain-containing transcription factors* (Supplementary Table S3).

JA and SA synthetase genes, such as *lipoxygenase*, *allene oxidase synthase*, *ACO3*, *AOC4*, *OPR3*, *OPR2*, *SAM:carboxyl methyltransferase*, and *jasmonic acid O-methyltransferase* (*JMT*), were extensively induced (Supplementary Table S3 at *JXB* online). Genes implicated in metabolism and signal transduction of other hormones, such as brassinosteroid, cytokinin, and gibberellin, were also altered (Supplementary Table S3). Taken together, the endogenous hormone level may have been altered by 2,4-D treatment.

### Transcriptional regulators involved in 2,4-D signal transduction

The data above indicated that TFs were involved in the 2,4-D signal transduction process (Fig. 4C). The data sets were compared with the 604 citrus TFs in planttfdb (http://planttfdb.cbi.edu.cn) (Zhang *et al.*, 2011) using BLASTN software (E-value <1E^–10^, identity ≥80%). On the Affymetrix genechip Citrus Genome Array, a total of 655 probe sets were tentatively annotated as putative TFs. Sixty-three of them were up-regulated by 2,4-D treatment and were distributed into 17 distinct TF families. They may serve as the regulator candidates ascribed to 2,4-D treatment (Supplementary Table S4 at *JXB* online). The largest three up-regulated TF families were AP2-ERF (19%), WRKY (17%), and NAC (13%). These TFs, together with the AS2 family (LBD, 6%) and the HB family (WOX, 3%), were over-represented (*P* < 0.05) (Fig. 4C). There were 11 members of the WRKY TF family (two *WRKY42* genes, *WRKY65*, *WRKY70*, *WRKY11*, three *WRKY33* genes, *WRKY41*, *WRKY30*, and an unknown WRKY domain-containing protein), eight NAC TFs (two *NAC28* genes, *NAC36*, and five *NAC domain containing proteins*), 11 ERFs, and an AP2 TF (five *ERF1* genes, *ERF12*, *DEAR2*, two *Rap2.6L* genes, *CBF2*, and *RAV2*) that were induced by 2,4-D treatment. NAC and WRKY TFs are the largest TF families in plants and they play important roles in stress defensive responses and the plant senescence process (Rushton *et al.*, 2010; Besseau *et al.*, 2012; Nakashima *et al.*, 2012). The up-regulation of these TFs may contribute to the transduction of defence signals and to regulation of the fruit senescent process.

### Stress-responsive genes were induced during the later periods (48 and 72 HPT)

The data above showed that stress-responsive genes were over-represented during the later periods (Fig. 4B). Interestingly, two-thirds of them were related to biotic stresses, and almost all of these biotic stress-responsive genes were up-regulated by 2,4-D, including: *chitinases*, *PR4*, *pathogenesis-related thaumatin family proteins*, *protease inhibitors*, and other pathogen or disease-related protein-encoding genes. In addition, putative wound-responsive proteins were also induced (Supplementary Table S5 at *JXB* online). The high expression of these stress-related genes may enhance fruit resistance to pathogen infections and enhance wound healing.

### Secondary metabolism-related genes were induced

**Fig. 5.**
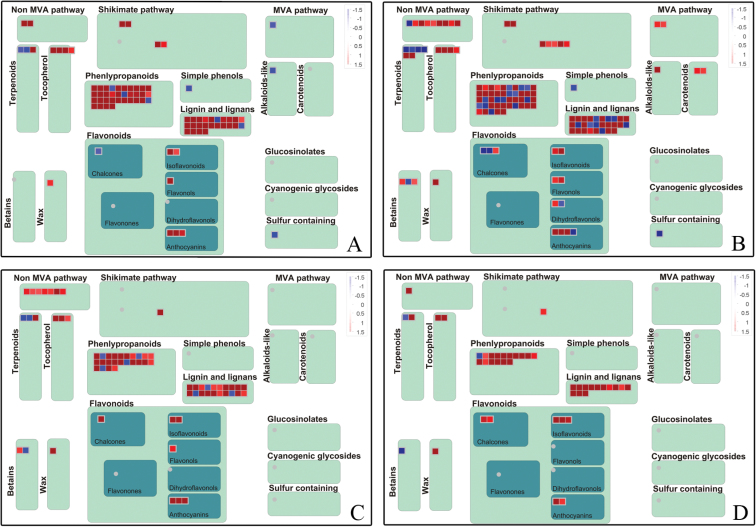
Schematic of the secondary metabolism pathway using the MapMan visualization platform. The red or blue squares indicate the up- or down-regulated genes involved in secondary metabolism pathways at 12 (A), 24 (B), 48 (C), and 72 HPT (D). (This figure is available in colour at *JXB* online.)

Secondary metabolism-related genes were consistently over-represented and up-regulated at the four time points. In order to visualize clearly the secondary metabolism pathways, these genes were imported onto the MapMan visualization platform (Fig. 5). The shikimate pathway and tocopherol biosynthesis pathways were significantly induced at 12 and 24 HPT. The non-melavonate (MVA) pathway for biosynthesis of isoprenoids was active at 24 and 48 HPT and the flavonoid pathway was active at all four time points.

Genes involved in phenylpropanoid metabolism and lignin production were very active at all four time points. Key enzymes involved in monolignol biosynthesis, such as *PAL1*, *PAL2*, *PAL4*, *4CL*, *CAD*, *CCoAOMT*, *COMT*, and putative peroxidases, were consistently induced (Supplementary Table S2 at *JXB* online). Interestingly, *CER1*, a gene encoding a key enzyme in the wax biosynthesis pathway, was consistently up-regulated. In addition, ABC transporters, such as *WBC11* and *CER5*, which are involved in the secretion of newly synthetized cuticular wax (Pighin *et al.*, 2004; Bird *et al.*, 2007), also significantly increased. Thus, it is possible that the high expression of wax and lignin biosynthesis-related genes in treated samples may result in high lignin and cuticle wax contents in the fruit pericarp.

### Comparative proteomics analysis of differentially accumulated proteins

**Fig. 6.**
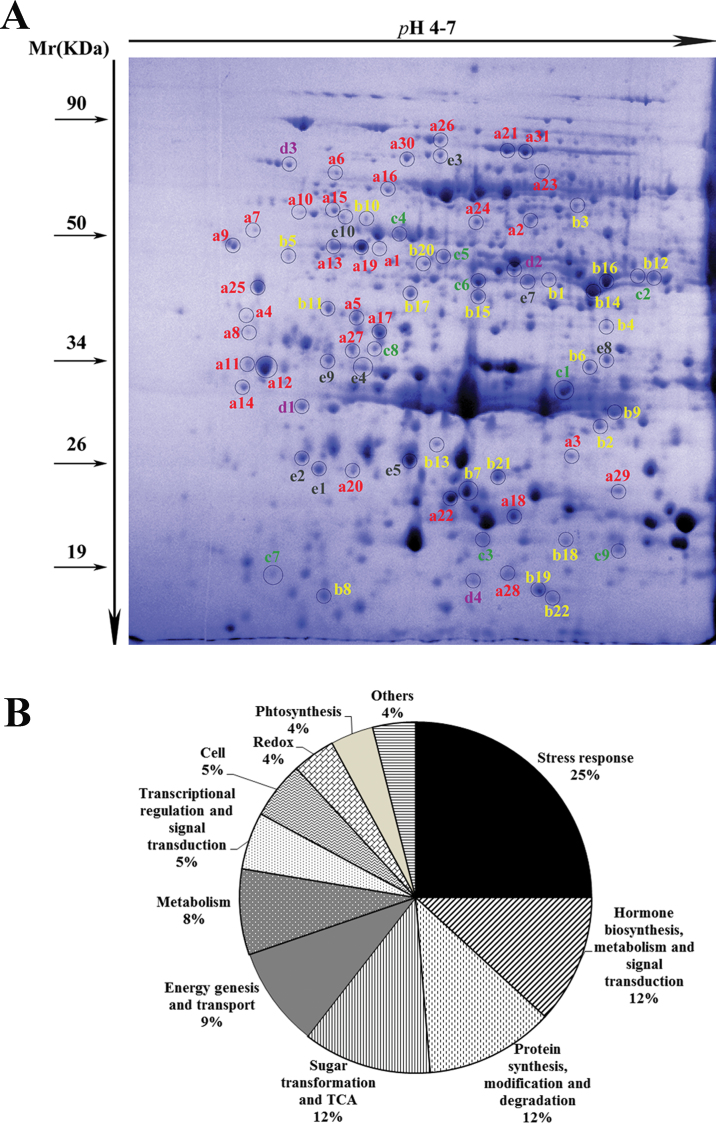
Differentially expressed proteins. (A) Image of a representative 2-D polyacrylamide gel. The spots in circles are the identified differentially expressed proteins. The letters a, b, c, d, and e represent five different expression modes: a represents proteins accumulated at one or more time points; b represents proteins that decreased at one or more time points; c represents proteins that accumulated at 12 or 24 HPT and then decreased at 48 or 72 HPT; d represents proteins that decreased at 12 or 24 HPT and then increased at 48 or 72 HPT; and e represents proteins with complex expression modes. (B) Functional categorization and frequencies of all identified proteins in response to 2,4-D treatment. (This figure is available in colour at *JXB* online.)

In order to confirm whether 2,4-D has similar effects on gene expression at the post-transcriptional level, total proteins in the control and treated fruit peel at 12, 24, 48, and 72 HPT were isolated and used for 2-D PAGE analysis. The comparative image analysis revealed that 619 protein spots showed reproducible staining patterns among the 27 gels. These proteins had isoelectric points between 4.0 and 7.0, and molecular masses ranging from 10kDa to 100kDa. In total, 99 spots showed significant changes in abundance (FC >2 and *P* < 0.01), and 76 of them were successfully identified by combined MS/MS (Fig. 6A).

The assignment of functional categories for the 76 proteins was based on the NCBI non-redundant protein expressed sequence tag (EST) database for green plants and on the Citrus EST database in order to enhance annotation reliability (Supplementary Table S6 at *JXB* online). Finally, these proteins were classified into 11 different functional groups (Fig. 6B). According to the transcriptional profile results, stress-responsive proteins (25%) and hormone-related proteins (12%) were the two largest classes of identified proteins. In addition, protein biosynthesis-, modification-, and degradation-related enzymes (12%) and sugar transformation- and tricarboxylic acid- (TCA) (12%) related proteins were also considerably affected by 2,4-D. BLASTP (e <10^–15^) was used to match the detected amino acid sequences of the differentially expressed proteins to the peptide chains that had been translated from the probe set ESTs on the microarray, and seven proteins were found with similar expression patterns at both the transcriptional and post-transcriptional levels (marked in bold in Supplementary Table S6).

#### 2,4-D significantly altered the expression of stress-responsive proteins

Three proteins involved in pathogen–plant interactions, chitinase (spot a25), miraculin-like protein 2 (spot a26), and osmotin 34 (spot d1), were induced by 2,4-D treatment. HSP abundances also changed. For example, HSP19 class II (spot c3), mitochondrial HSP23.5 (spots e1 and e2), and chaperonins (spot e3 and a30) were differentially accumulated, but HSA32 and HSP21 (spots b4 and e5) decreased after treatment. The different expression modes of these stress-responsive proteins may relate to their special functions in preserving fruits from various biotic and abiotic stresses during the storage period. C2 domain-containing proteins have Ca^2+^-dependent membrane-binding motifs and are involved in plant resistance to pathogen infection, and osmotic, dehydration, and oxidative stresses (Kim *et al.*, 2008; Yokotani *et al.*, 2009). In this study, C2 domain-containing proteins (spots a13, a1, and a19) and a calcium-binding EF hand family protein (spot a7) significantly accumulated after treatment. This indicated that calcium signalling may be involved in 2,4-D signal transduction and may promote defence responses in 2,4-D-treated fruits.

#### 2,4-D elevated the activities of hormone biosynthesis enzymes

The activities of enzymes involved in ethylene and JA biosynthesis were altered by 2,4-D treatment. *S*-Adenosylmethionine synthetase 1 (SAMS1) and SAMS2 (spots a2 and a24) in the ethylene synthesis pathway significantly accumulated after 2,4-D treatment. Allene oxide cyclase (AOC) is an important enzyme that catalyses the formation of *cis*-(+)-oxo-phytodieonic acid (OPDA; a direct precursor of JA). In this study, AOC (spot a3) significantly accumulated after 2,4-D treatment. It is worth noting that both SAMS and AOC were up-regulated at both the transcriptional and post-transcriptional levels, and this may lead to an increase in ethylene and JA.

#### Other proteins that are related to fruit ripening and senescence

Some proteins involved in fruit ripening and senescence were altered by 2,4-D treatment. Previous research has shown that cysteine proteases are involved in maintaining and stabilizing protein structure and function (González-Rábade *et al.*, 2011), plant senescence, and stress tolerance (Parrott *et al.*, 2010; Chen *et al.*, 2012). In this study, cysteine proteases (spots a12 and a11) increased, while cysteine protease inhibitor (spot b9) decreased after treatment. Proteins involved in fruit ripening and sensory quality were also identified, for example glycolysis/gluconeogenesis pathway proteins (putative phosphoglycerate mutase, spots a21 and a31; enolase, spot a16), malate dehydrogenase (spots e7, b1, and b14–b16), and succinyl-CoA synthetase (spot c5).

### Effect of 2,4-D on hormone metabolism and secondary metabolism

Both the transcript and protein analysis results suggested that hormone metabolism and secondary metabolism in Olinda orange rind changed after 2,4-D treatment. In order to verify this possibility further, the hormone and lignin contents were measured and the ultrastructure of the cuticle wax was observed.

#### Endogenous hormones levels were altered by 2,4-D

**Fig. 7.**
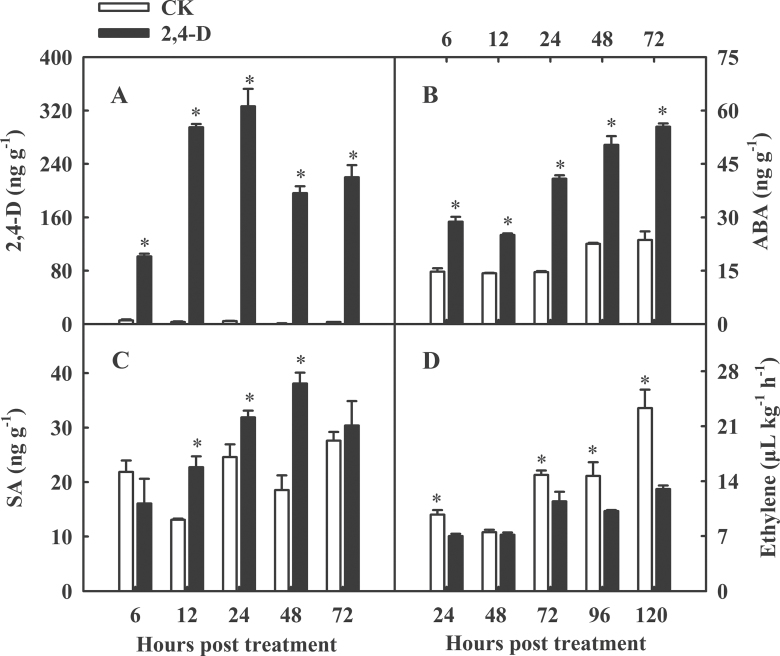
2,4-D residues (A), ABA (B), and SA (C) levels in Olinda fruit peel at 12, 24, 48, and 72 HPT, and ethylene production (D) of the whole fruit at 1, 2, 3, 4, and 5 DPT. * represents significance at *P* < 0.05.

After analysis, 2,4-D residues and endogenous hormones were detected in the fruit peel. The 2,4-D residue levels were highly elevated in the treated fruit peel. They rose from 101.6ng g^–1^ at 6 HPT to their highest levels of 326.5ng g^–1^ at 24 HPT, but then dropped to 200ng g^–1^ after 48 HPT whereafter they remained more or less constant (Fig. 7A). ABA contents also increased to about twice the levels seen in the control samples (Fig. 7B). SA significantly accumulated between 12 and 48 HPT (*P* < 0.05) (Fig. 7C). Ethylene production by 2,4-D-treated fruits declined (*P* < 0.05) by >20%, except at 2 DPT where there was a slight decrease (Fig. 7D). These data indicated that the levels of endogenous hormone in orange peel changed within a short period of time, accompanied by high levels of auxin (2,4-D), ABA, and SA, and low levels of ethylene release.

#### Effect of 2,4-D on lignin production in fruit peel

The acetyl bromide-soluble lignin contents in fruit peel were determined in order to confirm the inference that 2,4-D has a positive effect on lignin biosynthesis. The results showed that lignin contents slightly decreased in the control fruit peel, but increased in the treated fruit peel during storage (Fig. 1C). At 72 HPT, it was 14% higher in the treated fruit peel than in the control, and this gap rose to 48% after 1 month (*P* < 0.05) at 30 DPT. This result suggested that 2,4-D could effectively induce lignin accumulation.

#### Effect of 2,4-D on the ultrastructure of fruit epicuticular wax

**Fig. 8.**
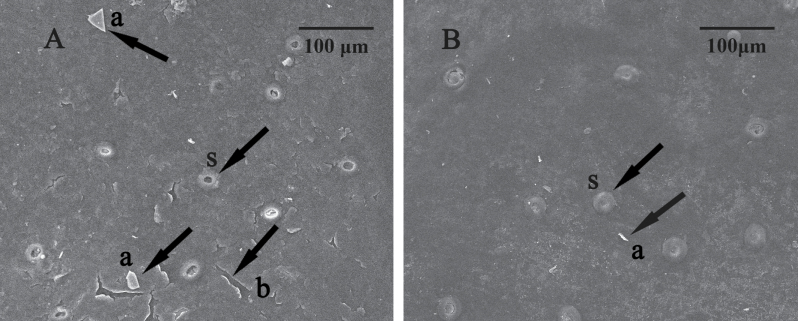
The ultrastructure of the control fruit surface (A) and the 2,4-D-treated Olinda orange fruit surface (B) observed by scanning electron microscopy. S, a, and b represent stomata, crystal wax, and cracks in the cuticular wax, respectively.

The microstructure of the fruit peel cuticular layer was examined by scanning electron microscopy. The surface of the control fruit was rough and had crystal waxes (Fig. 8A, a) and cracks (Fig. 8A, b) on it. The epicuticular wax was squamous and loosely covered the cuticle. The margins of the stomatal guard cells were obscured because they were heavily embedded within the cuticular wax, but the stomatal apertures could be clearly observed (Fig. 8A, S). In contrast, the treated fruit surface was relatively smooth (Fig. 8B) and the epicuticular wax tightly covered the cuticle. The cracks had disappeared and the volume of crystalline wax (Fig. 8B, a) had decreased. The stomatal apertures had swelled out and the pores were clogged by waxes (Fig. 8B, S).

### Effect of 2,4-D on the water content of fruit peel

The water contents were measured in the fruit peel during the storage period because the morphology changes in the epicuticular wax and stomata may have affected fruit peel water loss. Generally, the water content in the treated fruit peel was ~3% higher than in the control (Fig. 1D). Water content was maintained at ~72% in the control fruit peels, but was ~75% in the treated peels at both 72 HPT and 30 DPT. This result indicated that 2,4-D treatment could increase and maintain the water content in citrus fruit peel during the post-harvest storage period.

## Discussion

### 2,4-D altered the levels of endogenous hormones and delayed fruit senescence

The processes of fruit development, ripening, and senescence are determined by dynamic changes in hormone levels. When entering the ripening stage, the auxin content decreases and ethylene release or ABA biosynthesis gradually increases. Olinda orange fruit treated with 2,4-D has very high levels of auxin. 2,4-D levels rose to 101.6ng g^–1^ at 6 HPT, reached a maximum of 326.5ng g^–1^ at 24 HPT, and then remained at ~200ng g^–1^ after 48 HPT (Fig. 7A). The accumulation of 2,4-D within 24h after treatment was probably caused by two factors. First, it was actively transported into cells, but was not pumped out by efflux carriers, which could not use the 2,4-D as a substrate (Delbarre *et al.*, 1996). Secondly, GH3 subfamily family II members, such as *GH3.1*, which encode enzymes catalysing the formation of many inactive IAA–amino acid conjugates when plant cell auxin levels are high, have a low affinity for 2,4-D (Staswick *et al.*, 2005). It is worth noting that the expression of multidrug resistance transporter genes involved in plant detoxification and adaption to high levels of 2,4-D, such as pleiotropic drug resistance 6 (*PDR6*), *PDR11*, *PDR12*, multidrug resistance 4 (*MDR4*), *MDR17*, multidrug resistance protein 2 (*MRP2*), and *MRP5*, was mainly up-regulated at 24 HPT (Supplementary Table S2 at *JXB* online). These genes may be involved in extruding 2,4-D from cells and preventing fruit damage caused by the overaccumulation of 2,4-D. Consistently, the 2,4-D concentration reached its peak level at 24 HPT and then declined and reached an equilibrium at 48 HPT (Fig. 7A). In addition, *GH3* is one of the three families of early auxin-responsive genes that can be immediately induced by IAA or by synthetic auxins within a few minutes. The persistent induction of *GH3.1* indicated that 2,4-D had long-lasting rather than instantaneous effects.

At high concentrations, 2,4-D has generally been used as a herbicide to kill dicot weeds. It causes hormone level changes that are induced by the overaccumulation of 2,4-D in plant cells (Pazmiño *et al.*, 2012). Overaccumulation of 2,4-D activates ethylene overproduction and stimulates ABA biosynthesis. ABA accumulation causes stomatal closure and can induce overproduction of ROS, the main factor responsible for oxidative damage and cell death (Pazmiño *et al.*, 2011). In the citrus fruits post-harvest handling industry, 2,4-D has been used as an effective anti-stalling agent. In this study, the results showed that 2,4-D significantly reduced ethylene production (Fig. 7D) by the whole fruit, and induced the expression of *NCED3*, *NCED4*, and *NCED5* (Supplementary Table S3 at *JXB* online) and ABA production (Fig. 7B). Hence, it was inferred that the ethylene production decrease in 2,4-D-treated citrus fruit may retard fruit senescence during storage, but the exact mechanisms behind this still need to be explored. The accumulation of ABA in fruit peels might induce stomatal closure, reduce respiration (Fig. 1B), and inhibit the consumption of energy-producing compounds.

The defence hormone, SA, was induced after treatment (Fig. 7C). AOC catalyses the unstable allene oxide into *cis*-(+)-OPDA, which is the naturally occurring precursor of JA biosynthesis, and *AOC* significantly increased at the transcriptional (Supplementary Table S3 at *JXB* online) and proteomic levels (Supplementary Table S6, spot a3) in 2,4-D-treated fruit. Unfortunately, JA levels were too low to detect in the samples, which may be due to the fact that JA is usually induced by pathogen infection or mechanical injury.

In summary, the hormone levels were altered in Olinda orange fruit peel after 2,4-D treatment. In contrast to the 2,4-D herbicide mechanism, the high levels of auxin (2,4-D) reversed fruit senescence by inhibiting ethylene production and respiration and kept fruit metabolic activity low. Moreover, 2,4-D induced ABA and defence hormones, such as SA and JA, enhanced fruit defence against various stresses during storage, and reduced the incidence of fruit decay (Fig. 1A).

### 2,4-D treatment may enhance fruit defences against various stresses

Post-harvest fruit faces various biotic and abiotic stresses during storage. Water loss and pathogen infection are two of the most important factors that affect fruit commercial quality during long-term storage. In this study, fruit drenched with 2,4-D showed significant decreases in the incidence of decay (Fig. 1A) and a high water content in the peel (Fig. 1D). The ‘omics’ data revealed that the defence hormone, SA, accumulated in treated fruit peel, and many genes, proteins, and secondary metabolites downstream of the defence signalling pathway were also up-regulated by 2,4-D. TFs that participate in plant defence signalling, such as the WRKY, NAC, and AP2-EREBP family members (Zhang *et al.*, 2009; Li *et al.*, 2011), were also significantly up-regulated. The NAC and WRKY TFs are the largest TF families in plants. They are involved in the regulation of many different plant processes, such as biotic or abiotic stresses and plant senescence (Rushton *et al.*, 2010; Besseau *et al.*, 2012; Nakashima *et al.*, 2012). It was found that *WRKY11*, *WRKY30*, *WRKY33*, *WRKY41*, *WRKY42*, *WRKY65*, and *WRKY70* were differentially up-regulated during the earlier time periods (12 and 24 HPT). Studies in other species have shown that overexpression of *WRKY30* or *WRKY11* dramatically increased osmotic stress tolerance (Liu *et al.*, 2011; Shen *et al.*, 2012), and Ülker *et al.* (2007) reported that the *WRKY70* TF in *Arabidopsis* negatively influenced plant senescence and changed the defence signalling pathways. In addition, stress-responsive genes, especially for biotic stresses, were over-represented during the late time periods (48 and 72 HPT) (Fig. 4B). Moreover, a quarter of the 76 proteins with differential expression between the control and the treated samples were related to stress defence (Fig. 6B). For example, chitinases that were probably involved in hydrolysing the cell wall of fungal hyphae (Ballester *et al.*, 2010) were induced by 2,4-D at both the RNA (Supplementary Tablel S2 at *JXB* online) and protein levels (Fig. 6; Supplementary Table S6, spot a25). A gene encoding a member of the PR5 protein family (thaumatin-like protein) that was involved in membrane permeabilization, glucan hydrolysis, and apoptosis constantly increased after treatment. Correspondingly, the protease inhibitor, miraculin-like protein 2, which contains a thaumatin motif and has antifungal activity (Tsukuda *et al.*, 2006), was highly expressed at the protein level (Supplementary Table S6, spot a26). It is worth noting that miraculin-like protein and osmotin 34 (Supplementary Table S6, spot 1) were involved in plant tolerance to pathogen infections and resistance to water deficiency (Gimeno *et al.*, 2009; Viktorova *et al.*, 2012). HSPs play important roles in the re-establishment of cellular homeostasis by partially binding to the denatured proteins and preventing irreversible protein inactivation and aggregation under raised heat conditions. They are also involved in plant resistance. The present data show that 13 of the 32 HSPs, including HSP22.0, HSP21, HSP26.5-P, HSC70-5, HSP91, and DNAJ heat shock family proteins, were differentially expressed at the transcriptional level and were up-regulated at the proteome level at 24 and 72 HPT. Seven of the 19 stress response protein spots corresponded to HSPs, including five low molecular mass HSPs (Supplementary Table S6, spots c3, e1, e2, b4, and e5) and chaperonins HSP60 (Supplementary Table S6, spots e3 and e30). Previous studies have shown that chaperonins play an important role in assisting chloroplast proteins, such as Rubisco assembling proteins (Boston *et al.*, 1996). In this study, with the exception of chaperonins, Rubisco proteins (Supplementary Table S6, spot a10 and a15) also significantly accumulated in treated fruit peel, which might help explain the fact that 2,4-D could keep the fruit calyx green after prolonged storage. Generally, the expression changes in these defence genes and proteins will enhance fruit resistance to pathogen infection, and the fruit decay incidence results after 2 years of storage (Fig. 1A) further verified that 2,4-D enhanced the orange fruit antifungal activity.

The plant cuticle is a natural barrier against water loss (Domínguez *et al.*, 2011). When treated with 2,4-D, the crystalline waxes distributed on the fruit surface, which were dispersed on the control fruits, were partially melted, the cracks in the cuticle wax were sealed, and the wax cover on the cuticle became smoother and more condensed (Fig. 8B). The dense wax layer forms a protective shield that protects fruit from water evaporation, pathogen infection, and gas exchange across the cuticle and therefore inhibits water loss, fruit rot, and respiration. The changes in cuticle ultrastructure may have led to the high water content (Fig. 1D) seen in treated peel and to low levels of fruit rot.

## Conclusion

2,4-D-induced responses were a cascade process. First, exogenously applied 2,4-D altered the levels of endogenous phytohormones in the fruit peel, which induced ABA and SA production, but inhibited ethylene production. The induced expression of multiple TFs at an early stage amplified hormone signalling. As the cascade continued, defence-related genes and proteins, which play important roles in fruit adaption to the post-harvest environment and enhance fruit resistance to various stresses during storage, were expressed and began to accumulate. The final response was a reduction in respiration, which reduced energy consumption and fruit vitality. Changes in cuticle morphology reduced fruit water evaporation and pathogen infection, and the increases in water and lignin contents enhanced the mechanical strength of the fruit peel.

## Supplementary data

Supplementary data are available at *JXB* online.

Figure S1. Validation of the microarray expression data using quantitative real-time PCR.

Table S1. Annotational and functional classification of the genes contained in the eight different expression patterns.

Table S2. Expression, annotation, and categories of all 3413 genes that were differentially regulated by 2,4-D.

Table S3. Differential expression and annotations of hormone metabolism- and signal transduction-related genes.

Table S4. Expression, annotations, and classifications of the 63 up-regulated transcription factors.

Table S5. Differential expression and annotations of stress-responsive genes at 48 and 72 HPT.

Table S6. Identification of the differentially expressed proteins in the peel of Olinda orange fruit.

Supplementary Data
