# Supplementary material for: Comprehensive insights on how 2,4-dichlorophenoxyacetic acid retards senescence in post-harvest citrus fruits using transcriptomic and proteomic approaches
Source: J Exp Bot. 2013 Nov 8;65(1):61–74. doi: 10.1093/jxb/ert344 (PMC3883282; doi:10.1093/jxb/ert344)
Supplement: Supplementary Data [file supp_65_1_61__index.html]

Comprehensive insights on how 2,4-dichlorophenoxyacetic acid retards senescence in post-harvest citrus fruits using transcriptomic and proteomic approaches — Comprehensive insights on how 2,4-dichlorophenoxyacetic acid retards senescence in post-harvest citrus fruits using transcriptomic and proteomic approaches — Supplementary Data 

# Comprehensive insights on how 2,4-dichlorophenoxyacetic acid retards senescence in post-harvest citrus fruits using transcriptomic and proteomic approaches

## Supplementary Data

Data files

**Files in this Data Supplement:**

- Supplementary Data - Supplementary Data
- Supplementary Data - Supplementary Data
