# Supplementary material for: Comprehensive insights on how 2,4-dichlorophenoxyacetic acid retards senescence in post-harvest citrus fruits using transcriptomic and proteomic approaches
Source: J Exp Bot. 2013 Nov 8;65(1):61–74. doi: 10.1093/jxb/ert344 (PMC3883282; doi:10.1093/jxb/ert344)
Supplement: Supplementary Data [file supp_ert344_jexbot103507_file002.pdf]

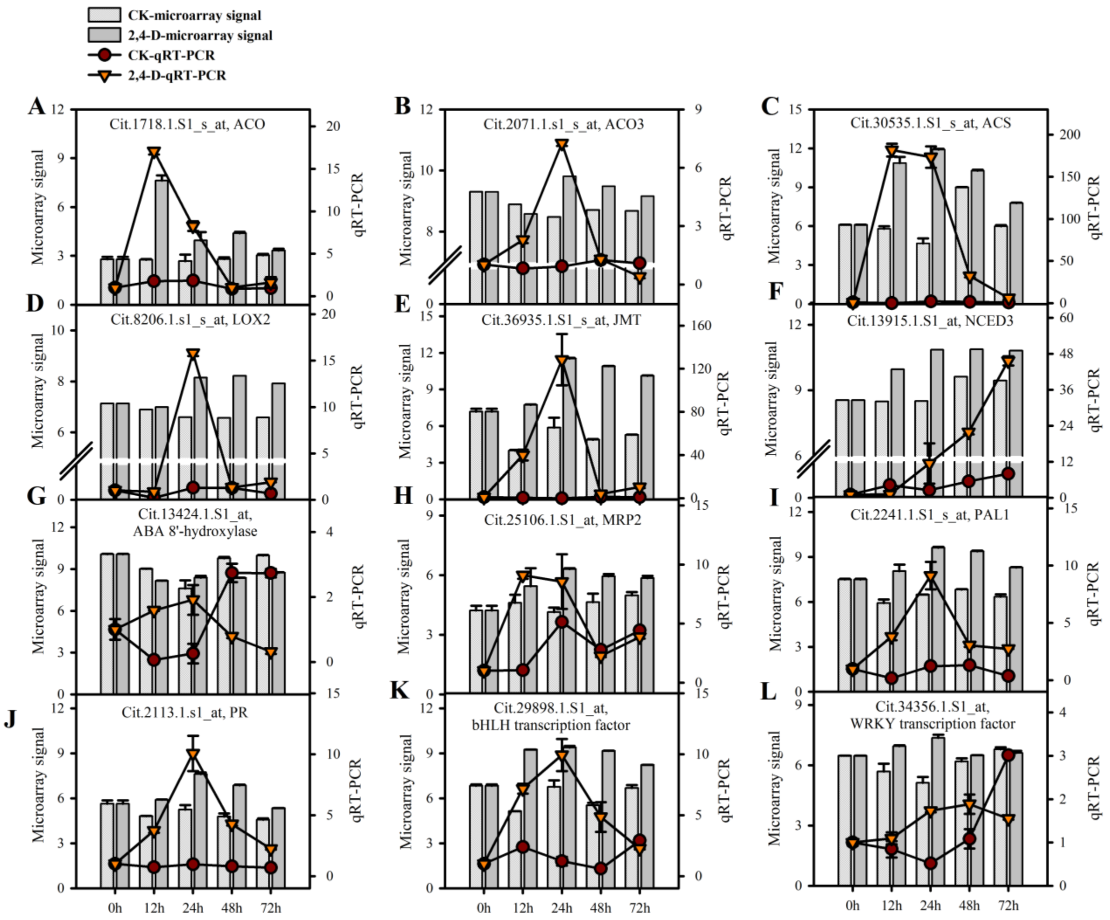

Supplemental Fig. 1. Validation of the microarray expression data using quantitative real-time PCR. Twelve interesting genes were selected: A is Cit.1718.1.S1_s_at, *ACO*; B is Cit.2071.1.s1_s_at, *ACO3*; C is Cit.30535.1.S1_s_at, *ACS*; D is Cit.8206.1.s1_s_at, *LOX2*; E is Cit.36935.1.S1_s_at, *JMT*; F is Cit.13915.1.S1_at, *NCED3*; G is Cit.13424.1.S1_at, *ABA 8'-hydroxylase*; H is Cit.25106.1.S1_at, *MRP2*; I is Cit.2241.1.S1_s_at, *PAL1*; J is Cit.2113.1.s1_at, *PR*; K is Cit.29898.1.S1_at, *basic helix-loop-helix transcription factor*; L is Cit.34356.1.S1_at, *WRKY* transcription factor.

Supplemental Table 1. Differential expressions and annotations of hormone metabolism and signal transduction related genes. Blank spaces indicate genes without change.

Probe set	Anotation	AGI	Log$_2$ ratio of treatment/CK			
			12 HPT	24 HPT	48 HPT	72 HPT
Auxin synthesis-degradation and signal transduction						
Cit.25129.1.S1_at	AFB5	AT5g49980		**1.19**		
Cit.12252.1.S1_at	GH3.1	AT2g14960	**1.71**	**2.41**	**2.56**	**2.39**
Cit.23036.1.S1_s_at	GH3.1	AT2g14960	**2.37**	**5.28**	**4.24**	**2.92**
Cit.5201.1.S1_at	GH3.1	AT2g14960	**1.90**	**4.48**	**3.80**	**2.70**
Cit.23488.1.S1_s_at	AGD2	AT1g60680	**-1.39**	**-1.87**	**-1.21**	**-1.25**
Cit.9559.1.S1_s_at	AGD2	AT1g60680		**-1.19**		
Cit.9560.1.S1_x_at	ATB2	AT1g60710		**1.40**		
Cit.24664.1.S1_at	Aldo/keto reductase family protein	AT1g60690	**-1.42**	**-1.96**		
Cit.11374.1.S1_s_at	IAR3	AT1g51760		**1.29**		
Cit.36875.1.S1_at	ILL2	AT5g56660		**1.16**		
Cit.12691.1.S1_s_at	JAR1	AT2g46370	**1.49**	**1.37**		
Cit.15048.1.S1_at	ARF4	AT5g60450	**-1.25**	**-1.37**		
Cit.15048.1.S1_s_at	ARF4	AT5g60450	**-1.10**			
Cit.14887.1.S1_at	ARF6	AT1g30330		**1.06**		
Cit.14051.1.S1_s_at	ARF10	AT2g28350			**1.09**	
Cit.17407.1.S1_at	IAA4	AT5g43700		**-1.16**		
Cit.5636.1.S1_s_at	IAA4	AT5g43700	**1.40**	**2.09**	**1.54**	
Cit.8972.1.S1_s_at	IAA16	AT5g43700	**-1.57**			
Cit.8403.1.S1_s_at	Similar to unknown protein	AT3g15450.1	**1.33**			
Cit.13706.1.S1_s_at	Similar to unknown protein	AT3g15450.1	**1.44**	**1.77**		
Cit.18006.1.S1_at	Similar to unknown protein	AT3g15450.1	**1.24**	**1.90**		
Cit.17330.1.S1_at	Auxin-responsive family protein	AT2g46690	**-1.56**	**-1.51**		
Cit.29704.1.S1_s_at	Auxin-responsive family protein	AT2g46690	**-1.15**		**-1.31**	

Cit.5615.1.S1_s_at	Auxin-responsive family protein	AT1g75590		**-1.07**	**-1.41**	
Cit.11105.1.S1_s_at	Auxin-responsive family protein	AT3g25290		**-1.27**		
Cit.11178.1.S1_s_at	Auxin-responsive family protein	AT5g35735		**1.02**		
Cit.10697.1.S1_s_at	Auxin-responsive family protein	AT5g20820	**1.59**	**1.17**		
Cit.15712.1.S1_at	Auxin-responsive family protein	AT1g72430	**2.37**	**1.06**	**1.32**	
ABA synthesis-degradation and signal transduction						
Cit.16305.1.S1_at	NCED4	AT4g19170		**-1.98**		
Cit.17235.1.S1_s_at	NCED5	AT1g30100	**2.25**	**1.94**		
Cit.29734.1.S1_s_at	NCED3	AT3g14440	**2.03**	**2.10**		
Cit.8156.1.S1_at	NCED4	AT4g19170	**2.17**	**1.21**	**1.32**	
Cit.13915.1.S1_at	NCED3	AT3g14440	**1.47**	**2.34**	**1.26**	**1.37**
Cit.3748.1.S1_s_at	ABF4	AT3g19290		**-1.04**		
Cit.15430.1.S1_at	PP2C family protein	AT3g12620		**1.37**		
Cit.31326.1.S1_at	PP2C, putative	AT3g62260		**1.18**		
Cit.29625.1.S1_at	PP2C, putative	AT2g30020	**-1.81**	**1.57**	**-1.55**	
Cit.12329.1.S1_at	PP2C, putative	AT5g53140		**-1.44**	**-1.25**	
Cit.12329.1.S1_s_at	PP2C, putative	AT5g53140		**-1.49**	**-1.22**	
Cit.31435.1.S1_at	PP2C, putative	AT5g53140		**-1.06**	**-1.10**	
Cit.4061.1.S1_s_at	PP2C, putative	AT1g07160	**-1.34**		**-1.40**	
Cit.17914.1.S1_at	PP2C-related	AT1g32640		**-1.04**		
Cit.15850.1.S1_at	SNRK2.6	AT4g33950	**1.02**	**1.24**		
Cit.13263.1.S1_s_at	HVA22E	AT5g50720		**-1.60**		
Cit.3274.1.S1_s_at	HVA22 family protein	AT5g42560	**-1.18**	**-1.10**		
Cit.34429.1.S1_s_at	GRAM domain-containing protein	AT5g23370		**1.14**		
Ethylene synthesis-degradation and signal transduction						
Cit.4491.1.S1_at	ACC synthase ACS6	AT4g11280		**1.21**		
Cit.18037.1.S1_at	ACC synthase ACS1	AT3g61510	**1.92**	**4.36**	**1.15**	**2.03**
Cit.30535.1.S1_at	ACC oxidase ACO4	AT1g05010		**1.14**		
Cit.21723.1.S1_s_at	ACC oxidase ACO4	AT1g05010	**4.58**	**6.74**	**1.18**	**1.64**
Cit.30535.1.S1_s_at	ACC oxidase ACO4	AT1g05010	**5.07**	**7.23**	**1.26**	**1.80**

Cit.20188.1.S1_at	2OG-Fe(II) oxygenase family protein	AT1g49390	**-1.56**	**-2.68**	**-1.29**	**-1.00**
Cit.23448.1.S1_s_at	2OG-Fe(II) oxygenase family protein	AT1g49390	**-1.66**	**-2.61**	**-1.33**	
Cit.23448.1.S1_x_at	2OG-Fe(II) oxygenase family protein	AT1g49390	**-1.38**	**-1.56**		
Cit.11435.1.S1_s_at	2OG-Fe(II) oxygenase family protein	AT3g19000			**1.22**	**1.50**
Cit.6827.1.S1_x_at	2OG-Fe(II) oxygenase family protein	AT1g17010		**1.32**		
Cit.5281.1.S1_at	2OG-Fe(II) oxygenase family protein	AT5g05600	**1.82**	**2.18**		**1.32**
Cit.13128.1.S1_s_at	2OG-Fe(II) oxygenase family protein	AT5g24530	**1.18**	**1.37**	**2.30**	
Cit.18154.1.S1_at	2OG-Fe(II) oxygenase family protein	AT5g20400	**2.81**	**1.39**	**1.32**	
Cit.15355.1.S1_at	2OG-Fe(II) oxygenase family protein	AT4g10490	**2.06**	**2.09**	**1.87**	**1.41**
Cit.5282.1.S1_at	2OG-Fe(II) oxygenase family protein	AT5g05600	**1.93**	**2.96**	**1.31**	**1.70**
Cit.5316.1.S1_at	2OG-Fe(II) oxygenase family protein	AT5g05600	**4.96**	**6.35**	**5.80**	**3.43**
Cit.14031.1.S1_at	ERS1	AT2g40940	**1.03**			
Cit.28131.1.S1_s_at	ETR2	AT3g23150	**2.24**	**2.48**		
Cit.22763.1.S1_s_at	ERF1	AT3g23240	**4.56**	**4.12**	**-1.52**	
Cit.4810.1.S1_at	ERF1	AT3g23240	**3.52**	**3.65**	**-2.54**	
Cit.5984.1.S1_at	ERF1	AT3g23240	**3.10**	**2.39**		
Cit.16636.1.S1_at	ERF2	AT5g47220	**1.83**	**1.80**	**1.15**	
Cit.5891.1.S1_at	ERF2	AT5g47220	**1.56**	**2.02**	**-1.72**	
Cit.29533.1.S1_s_at	ERF6	AT4g17490	**-1.01**		**-1.26**	
Cit.14141.1.S1_s_at	ERF7	AT3g20310			**-1.08**	
Cit.11704.1.S1_s_at	ERF9	AT5g44210			**-1.17**	
Cit.17142.1.S1_s_at	ERF9	AT5g44210			**-1.35**	
Cit.16845.1.S1_at	ERF12	AT1g28360	**1.52**	**1.46**		
Cit.15228.1.S1_at	ERF13	AT2g44840		**-1.31**		**-1.02**
Cit.2675.1.S1_s_at	AP2 domain-containing transcription factor, putative	AT5g51190		**1.19**		
Cit.14303.1.S1_at	AP2 domain-containing transcription factor	AT5g61590	**1.44**	-0.67	**-1.95**	
Cit.22941.1.S1_at	Similar to unknown protein	AT2g25190.1		**-1.20**		
Cit.14104.1.S1_at	Similar to unknown protein	AT2g25190.1		**-1.43**	**-1.40**	
Cit.14104.1.S1_s_at	Similar to unknown protein	AT2g25190.1		**-1.36**	**-1.18**	
Cit.14104.1.S1_x_at	Similar to unknown protein	AT2g25190.1		**-1.59**	**-1.23**	

Cit.18140.1.S1_at	Universal stress protein family protein	AT3g62550		**-1.06**		
Cit.5117.1.S1_at	Universal stress protein family protein	AT3g62550	**1.06**	**1.28**		
JA synthesis-degradation and signal transduction						
Cit.9904.1.S1_s_at	Lipoxygenase LOX2	AT3g45140			**1.55**	
Cit.4573.1.S1_at	Lipoxygenase LOX2	AT3g45140		**2.17**	**1.44**	
Cit.8206.1.S1_s_at	Lipoxygenase LOX2	AT3g45140		**1.55**	**1.66**	**1.33**
Cit.12474.1.S1_s_at	Lipoxygenase, putative	AT1g72520		**1.13**		
Cit.18017.1.S1_s_at	Lipoxygenase, putative	AT1g72520		**1.32**		
Cit.10444.1.S1_at	CYP74B2	AT4g15440		**1.73**	**1.41**	
Cit.10979.1.S1_at	Allene oxide cyclase AOC4	AT1g13280			**1.01**	
Cit.2071.1.S1_s_at	Allene oxide cyclase AOC3	AT3g25780		**1.33**		
Cit.14108.1.S1_at	12-Oxo-PDA-reductase OPR3	AT2g06050		**1.29**		
Cit.10684.1.S1_at	12-Oxo-PDA-reductase OPR2	AT1g76690	**3.69**	**4.37**		
Cit.10685.1.S1_s_at	12-Oxo-PDA-reductase OPR2	AT1g76690	**2.64**	**3.59**	**1.04**	
Cit.30497.1.S1_at	JMT	AT1g19640		**2.02**	**2.12**	**1.07**
Cit.11410.1.S1_s_at	Kelch repeat-containing protein	AT3g07720		**-1.36**		
SA synthesis-degradation and signal transduction						
Cit.12979.1.S1_at	SAM:carboxyl methyltransferase family protein	AT5g66430	**1.09**			
Cit.36935.1.S1_s_at	JMT	AT1g19640	**3.75**	**5.65**	**6.03**	**4.83**
Other hornome related genes						
Cit.14413.1.S1_at	DET2	AT5g16010	**1.18**	**-1.91**		
Cit.26842.1.S1_at	DWF1	AT3g19820	**-1.51**	**-1.96**		
Cit.33172.1.S1_at	DWF4	AT3g50660		**1.25**		
Cit.26526.1.S1_at	CYP90D1	AT3g13730		**1.06**		
Cit.10659.1.S1_s_at	Sterol methyltransferase SMT1	AT5g13710		**-1.05**		
Cit.10660.1.S1_at	Sterol methyltransferase SMT1	AT5g13710		**-1.53**		
Cit.22644.1.S1_at	Isopentenyltransferase IPT3	AT3g63110			**-1.06**	
Cit.18362.1.S1_at	Cytokinin oxidase CKX5	AT1g75450	**1.02**	**1.27**		
Cit.17149.1.S1_s_at	Histidine kinase 1	AT2g17820		**-1.19**		

Cit.32142.1.S1_at	Histidine kinase CKI1	AT2g47430	**-1.57**			
Cit.25357.1.S1_at	GA2OX8 (gibberellin 2-oxidase 8)	AT4g21200			**1.03**	
Cit.18766.1.S1_at	GA2OX2	AT1g30040	**1.54**			
Cit.25361.1.S1_s_at	Scarecrow-like transcription factor SCL8	AT5g52510		**-1.14**		
Cit.10032.1.S1_x_at	GAST1 protein homolog GASA1	AT1g75750				**-1.41**
Cit.35768.1.S1_s_at	GAST1 protein homolog GASA5	AT3g02885			**1.99**	
Cit.11064.1.S1_at	GAST1 protein homolog GASA4	AT5g15230			**1.07**	**1.14**
Cit.9890.1.S1_s_at	Gibberellin-regulated family protein	AT2g14900			**-2.11**	**-1.26**
Cit.30950.1.S1_at	Gibberellin-regulated family protein	AT2g30810		**1.27**		

Supplemental Table S2. Differential expressions and annotations of stress-responsive genes at 48 and 72 HPT.

**Probe set**	**Anotation**	**AGI**	**Log_2 ratio of treatment/CK**	
			**48 HPT**	**72 HPT**
Biotic stress related genes				
Cit.14913.1.S1_s_at	Acidic endochitinase (CHIB1)	AT5g24090	**1.51**	**1.35**
Cit.20412.1.S1_s_at	CHI-B	AT3g12500	**2.00**	**1.55**
Cit.20412.1.S1_s_at	CHI-B	AT3g12500	**2.00**	**1.55**
Cit.10594.1.S1_at	Chitinase (CHI) class IV	AT3g54420		**1.41**
Cit.15242.1.S1_at	Chitinase (CHI) class IV	AT3g54420	**2.07**	**3.03**
Cit.39297.1.S1_at	Disease resistance family protein	AT1g47890	**1.34**	
Cit.25559.1.S1_at	Disease resistance protein (CC-NBS-LRR class), putative	AT1g58410	**1.22**	
Cit.12682.1.S1_at	Disease resistance protein (NBS-LRR class), putative	AT3g14460	**-1.29**	
Cit.33610.1.S1_at	Disease resistance-responsive protein-related	AT1g58170	**1.69**	**1.17**
Cit.30907.1.S1_at	Glycosyl hydrolase family 19 protein	AT4g19800	**1.51**	**1.33**
Cit.27793.1.S1_at	Leucine-rich repeat family protein	AT1g74190	**1.46**	**1.07**
Cit.30114.1.S1_at	Leucine-rich repeat family protein	AT1g74180	**-1.51**	**-1.15**
Cit.15404.1.S1_at	Pathogenesis-related protein, putative	AT4g33720	**6.02**	**5.52**
Cit.11548.1.S1_at	Pathogenesis-related thaumatin family protein	AT1g20030	**2.43**	**3.55**
Cit.12699.1.S1_s_at	Pathogenesis-related thaumatin family protein	AT1g20030	**-1.06**	
Cit.753.1.S1_x_at	PR4	AT3g04720	**2.06**	**2.26**
Cit.21717.1.S1_at	PR4	AT3g04720	**1.29**	**1.02**
Cit.22589.1.S1_s_at	Protease inhibitor, putative	AT2g38870	**2.45**	**3.30**
Cit.8464.1.S1_s_at	Protease inhibitor, putative	AT2g38870	**1.04**	**1.47**
Cit.6364.1.S1_s_at	Secretory protein, putative	AT2g15220	**1.36**	**1.11**
Cit.21195.1.S1_at	Serine protease inhibitor	AT2g38870		**1.27**
Cit.19521.1.S1_s_at	Transferase family protein	AT5g17540	**1.09**	
Cit.31057.1.S1_s_at	Transferase family protein	AT5g17540	**2.86**	

Cit.16882.1.S1_x_at	Trypsin and protease inhibitor family protein	AT1g17860	**1.22**	
Cit.16962.1.S1_x_at	Trypsin and protease inhibitor family protein	AT1g17860	**1.41**	**1.38**
Cit.19362.1.S1_x_at	Trypsin and protease inhibitor family protein	AT1g17860	**1.27**	
Cit.22421.1.S1_x_at	Trypsin and protease inhibitor family protein	AT1g17860	**1.89**	**1.93**
Cit.29321.1.S1_x_at	Trypsin and protease inhibitor family protein	AT1g17860	**2.56**	**3.59**
Cit.29325.1.S1_x_at	Trypsin and protease inhibitor family protein	AT1g17860	**1.23**	
Cit.29356.1.S1_x_at	Trypsin and protease inhibitor family protein	AT1g17860	**2.64**	**4.22**
Cit.29368.1.S1_x_at	Trypsin and protease inhibitor family protein	AT1g17860	**2.27**	**4.12**
Cit.29373.1.S1_s_at	Trypsin and protease inhibitor family protein	AT1g17860	**1.73**	**2.69**
Cit.35442.1.S1_x_at	Trypsin and protease inhibitor family protein	AT1g17860	**2.11**	**4.85**
Abiotic stress related genes				
Cit.15338.1.S1_at	HSP17.4	AT3g46230	**-3.11**	**-2.13**
Cit.12593.1.S1_at	HSP26.5-P	AT1g52560	**-1.46**	**-1.25**
Cit.26433.1.S1_s_at	HSP26.5-P	AT1g52560	**-1.59**	**-1.16**
Cit.13558.1.S1_s_at	HSP70B	AT1g16030	**-1.15**	
Cit.35378.1.S1_s_at	DNAJ heat shock family protein	AT2g20560	**-1.16**	**-1.05**
Cit.13005.1.S1_at	GRP2B (glycine-rich protein 2B)	AT2g21060	**-1.17**	
Cit.30437.1.S1_s_at	RCI2B (rare-cold-inducible 2B)	AT3g05890	**-1.30**	
Cit.8878.1.S1_at	Enoyl-CoA hydratase/isomerase family protein	AT1g06550		**1.09**
Cit.8648.1.S1_at	Germin-like protein, putative	AT5g39120		**1.11**
Cit.29439.1.S1_at	wound-responsive family protein	AT4g10270		**1.52**
Cit.29721.1.S1_at	wound-responsive family protein	AT4g10270	**1.69**	**2.58**
Cit.5237.1.S1_s_at	wound-responsive family protein	AT4g10270	**1.47**	**2.61**
Cit.10014.1.S1_s_at	wound-responsive family protein	AT4g10270	**1.31**	**1.87**

Supplementary Table 3. Identification of the differentially expressed proteins in peel of Olinda orange fruit. The bold rows represent the proteins co-expressed at both transcriptional and posttranscriptional levels with similar patterns. Letters of a, b, c, d, and e represent five different expression modes: a, proteins accumulated at one or more time points of 12, 24, 48 and 72 HPT; b, proteins decreased at one or more time points; c, proteins accumulated at 12 or 24 HPT then decreased at 48 or 72 HPT; d, proteins decreased at 12 or 24 HPT then accumulated at 48 or 72 HPT; e, proteins with complex expression modes.

**Spot**	**Accession number**	**Annotation**	**Species**	**Theo. Mr (kD)/p*I***	**Exp. Mr (kD)/p*I***	**Protein score**	**Protein score C.I.%**	**Expression Changes (0h-12h-24h-48h-72h)**
**Stress response**								
d1	Gi|218830719	Osmotin 34	*Citrus clementina*	21.24/5.85	25.57/4.84	171	100	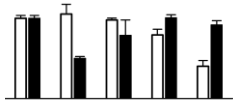
a25	Gi|63074640	Chitinase	*Citrus clementina*	12.58/5.13	41.58/4.54	82	98.10	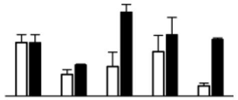
**a26**	**Gi|87299377**	**Miraculin-like protein 2**	***Citrus jambhiri***	**24.45/5.61**	**74.65/5.55**	**121**	**100**	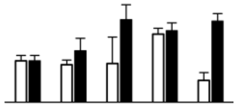
c3	Gi|30575570	HSP19 class II	*Citrus* X *paradisi*	11.19/ 8.01	15.47/5.82	197	100	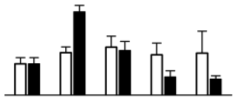

e1	Gi|188307148	Mitochondrial HSP23.5	*Citrus reticulata*	32.43/9.53	19.81/4.93	284	100	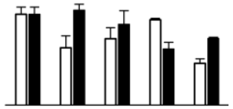
e2	Gi|218812876	Mitochondrial HSP23.5	*Citrus medica*	23.98/5.71	20.84/4.84	130	100	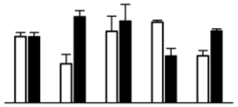
b4	Gi|46212644	HSA32	*Citrus sinensis*	30.65/5.69	35.61/6.45	117	100	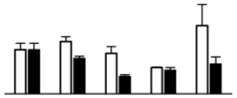
e5	Gi|63104305	HSP21	*Citrus* X *paradisi*	33.20/8.39	20.86/5.41	483	100	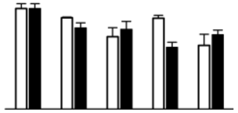
e3	Gi|16221	Chaperonin HSP60	*Arabidopsis thaliana*	61.65/ 5.66	69.43/5.56	264	100	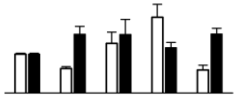
a30	Gi|2506277	Chaperonin 60 beta	*Pisum sativum*	63.29/5.85	68.70/5.37	398	100	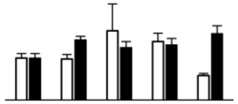
a13	Gi|188252573	C2 domain containing protein	*Citrus sinensis*	31.71/8.72	49.11/5.02	230	100	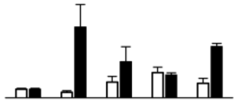
a1	Gi|218791092	C2 domain containing protein	*Citrus clementina*	30.43/5.10	48.14/5.24	151	100	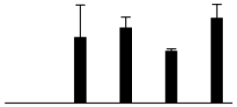

a19	Gi|31672073	C2 domain containing protein	*Citrus trifoliata*	27.51/5.97	48.90/5.15	236	100	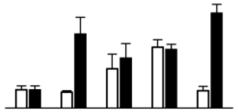
a29	Gi|55289848	Glycine rich protein 2	*Citrus trifoliata*	25.33/6.34	18.64/6.51	196	100	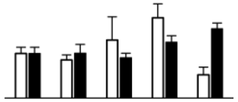
c1	Gi|188289622	Germin-like protein, putative	*Citrus sinensis*	34.15/9.23	28.05/6.22	186	100	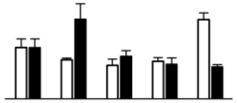
b7	Gi|116643152	Stress-related protein	*Citrus sinensis*	17.59 /5.67	18.58/5.74	315	100	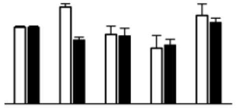
b11	Gi|28617273	B-1,3-glucanase 1	*Citrus Sinensis*	30.59/5.44	37.12/4.98	155	100	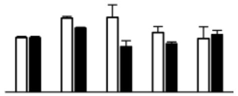
b5	Gi|218839004	Constitutive disease resistance 1	*Citrus clementina*	32.69/7.41	46.83/4.74	223	100	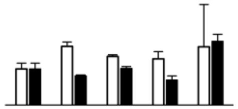
**b8**	**Gi|209925686**	**Responsive to dehydration 21**	***Citrus unshiu***	**21.36/6.08**	**12.37/4.98**	**165**	**100**	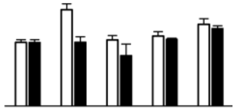
**Hormone biosynthesis, metabolism and signal transduction**								

**a2**	**Gi|56588757**	**S-adenosylmethionine synthetase 2**	***Citrus sinensis***	**30.30/9.06**	**53.96/6.03**	**262**	**100**	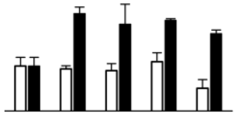
**a24**	**Gi|75308025**	**S-adenosylmethionine synthetase 1**	***Elaeagnus umbellata***	**43.56/ 5.50**	**53.44/5.80**	**269**	**100**	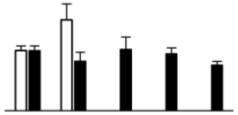
b12	Gi|728744	Auxin-induced protein PCNT115	*Nicotiana tabacum*	34.29/7.1	44.62/6.67	139	100	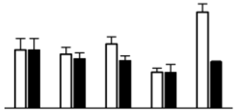
b6	Gi|270233805	Auxin induced unkonwn protein	*Vitis vinfera*	16.39/5.78	30.93/6.36	181	100	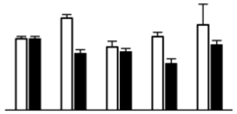
b13	Gi|55398044	ARF-GAP domain 2	*Citrus sinensis*	24.29/8.2	22.40/5.56	173	100	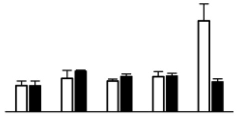
d2	Gi|188269048	Aldo/keto reductase family protein	*Citrus sinensis*	31.23/6.49	44.88/5.94	408	100	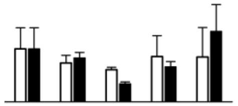
c2	Gi|62526573	Aldo/keto reductase family protein	*Manihot esculenta*	38.02/ 6.38	44.28/6.61	246	100	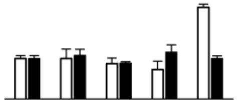
**a3**	**Gi|61808301**	**Allene oxide cyclase**	***Camptotheca acuminata***	**27.06/8.71**	**21.41/6.26**	**86**	**99.80**	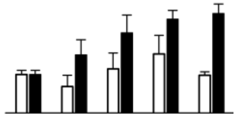

c4	Gi|31670062	Agmatine deiminase	*Citrus trifoliata*	31.17 /7.16	50.26/5.35	177	100	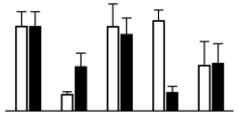
**Protein synthesis and degradation**								
a18	Gi|13094963	Eukaryotic elongation factor 5A-1	*Manihot esculenta*	17.82/ 5.6	16.74/5.97	130	100	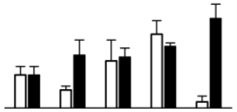
a4	Gi|188298374	Elongation factor 1-beta, putative	*Citrus sinensis*	31.97/7.71	36.79/4.42	166	100	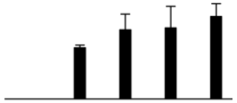
a28	Gi|28618533	Protein kinase C inhibitor, putative	*Citrus sinensis*	23.04/9.08	13.58/5.94	118	100	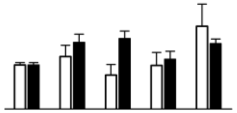
e6	Gi|34521947	Ubiquitin family protein	*Citrus sinensis*	20.31/4.72	67.90/4.75	119	100	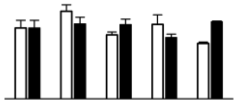
d3	Gi|110885389	Ubiquitin family protein	*Citrus clementina*	43.67/7.29	69.02/4.55	137	100	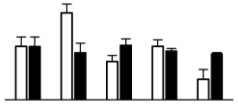
a14	Gi|225441983	20S proteasome alpha subunit E2	*Vitis vinifera*	26.13/ 4.65	28.09/4.40	274	100	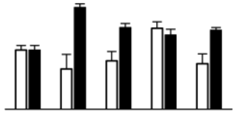

a12	Gi|188410065	Cysteine protease, putative	*Poncirus trifoliata*	35.52/6.7	30.60/4.57	136	100	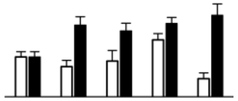
a11	Gi|188334808	Cysteine protease, putative	*Citrus sinensis*	29.94/8.36	31.26/4.43	110	99.99	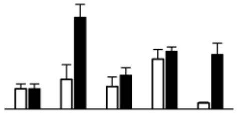
b9	Gi|188278038	Cysteine protease inhibitor, putative	*Citrus trifoliata*	34.43/6.77	25.75/6.48	409	100	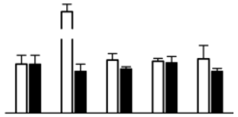
**Sugar transformation and TCA**								
a21	Gi|56528920	Phosphoglycerate mutase, putative	*Citrus sinensis*	32.18/6.79	71.67/5.90	290	100	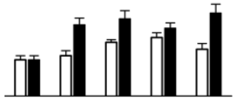
a31	Gi|188354130	Phosphoglycerate mutase, putative	*Citrus sinensis*	32.85//5.69	71.88/5.99	327	100	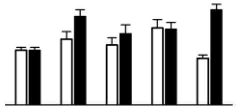
a16	Gi|188328322	Enolase	*Citrus reticulata*	31.61/9.41	61.27/5.28	179	100	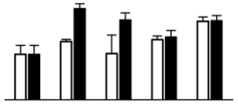
e7	Gi|125561648	NAD-dependent malate dehydrogenase	*Oryza sativa Indica Group*	41.77/7.66	43.36/6.03	115	100	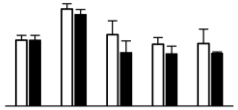

b1	Gi|218786344	Malate dehydrogenase (NAD), mitochondrial, putative	*Citrus clementina*	29.86/9.48	43.42/6.14	119	100	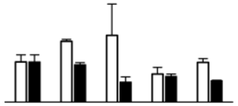
b14	Gi|110861996	Malate dehydrogenase (NAD), mitochondrial, putative	*Citrus clementina*	36.78/9.47	42.27/6.37	113	99.99	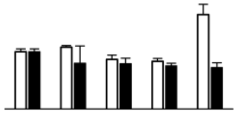
b15	Gi|114479586	Malate dehydrogenase	*Citrus junos*	43.67/6.91	39.46/5.76	360	100	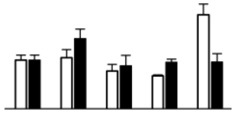
b16	Gi|225453490	Malate dehydrogenase, cytosolic, putative	*Vitis vinifera*	36.24/6.4	43.89/6.44	141	100	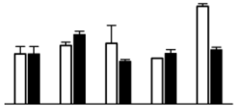
c5	Gi|226510248	Succinyl-coa synthetase B chain	*Zea mays*	45.51/ 5.99	46.41/5.58	124	100	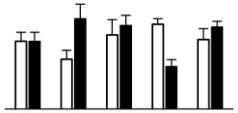
**Metabolism**								
b2	Gi|225461209	Flavodoxin-like quinone reductase 1	*Vitis vinifera*	21.73/ 5.83	24.71/6.42	177	100	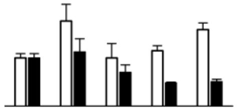
**a23**	**Gi|188321731**	**3-phosphoglycerate dehydrogenase**	***Citrus reticulata***	**28.67/10.14**	**65.93/6.09**	**183**	**100**	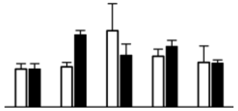

b17	Gi|188314872	O-acetylserine (thiol) lyase isoform C	*Citrus sinensis*	34.83/10.47	39.95/5.40	212	100	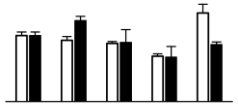
b18	Gi|224121906	Nucleoside diphosphate kinase 2	*Populus trichocarpa*	17.02/ 6.17	15.32/6.24	121	100	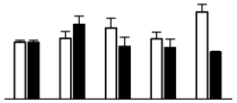
b19	Gi|63060347	Nucleoside diphosphate kinase 1	*Citrus clementina*	26.49/9.46	12.92/6.11	241	100	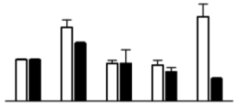
a5	Gi|115472485	Thiazole biosynthetic enzyme, chloroplast precursor (ARA6)	*Oryza sativa*	37.43/5.44	36.35/5.12	114	100	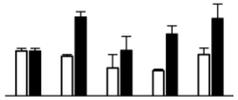
**Energy genesis and transport**								
e8	Gi|255538692	Gamma Carbonicanhydrase 1	*Ricinus communis*	29.62/ 5.73	31.64/6.45	128	100	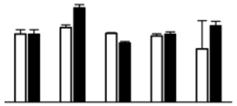
a20	Gi|192910736	ATP synthase D chain, mitochondrial	*Elaeis guineensis*	19.71/ 5.13	19.32/5.12	111	100	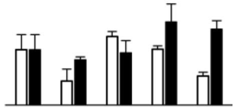
a6	Gi|188347714	Vacuolar ATP synthase subunit B	*Citrus sinensis*	34.76/6.19	65.15/5.02	286	100	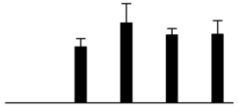

b20	Gi|12585490	Vacuolar ATP synthase subunit A	*Citrus unshiu*	68.28/ 5.29	45.43/5.48	674	100	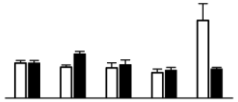
a22	Gi|188251304	Atpase-like protein	*Citrus sinensis*	32.31/9.05	18.03/5.65	220	100	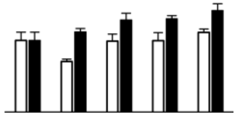
e9	Gi|255554349	6-phosphogluconolactonase	*Ricinus communis*	28.11/ 5.44	30.88/4.98	115	100	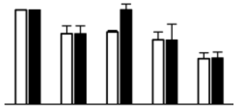
a27	Gi|61689787	6-phosphogluconolactonase	*Citrus sinensis*	21.88/7.63	32.39/5.11	171	100	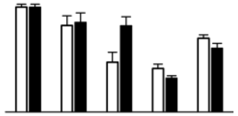
**Transcriptional regulation and signal transduction**								
a17	Gi|110885062	Remorin family protein	*Citrus clementina*	35.102/6.74	34.50/5.25	247	100	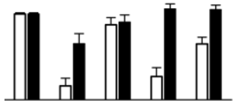
b3	Gi|224078632	RNA-binding protein 45, putative	*Populus trichocarpa*	42.55/ 5.91	58.11/6.27	139	100	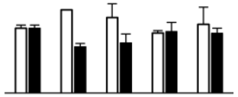
d4	Gi|242089407	Acetylglutamate kinase regulator	*Sorghum bicolor*	21.67/9.82	13.10/5.78	110	100	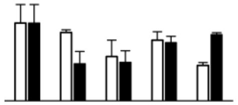

**a7**	**Gi|218803120**	**Calcium-binding EF hand family protein**	***Citrus clementina***	**25.37/4.97**	**51.79/4.51**	**113**	**99.99**	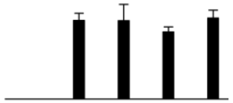
**Cell organization/ division/cycle**								
a8	Gi|62900641	Plastid-lipid-associated protein, chloroplastic	*Citrus unshiu*	35.25/ 5.24	35.24/4.38	337	100	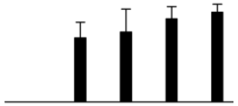
c6	Gi|55933692	Annexin	*Citrus sinensis*	32.43/5.62	41.93/5.77	322	100	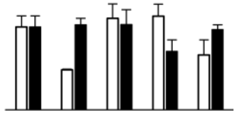
b10	Gi|255565619	Cell division protein ftsz	*Ricinus communis*	52.55/6.73	54.10/5.17	148	100	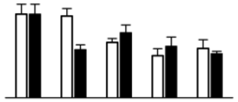
b21	Gi|188396422	Peptidyl-prolyl cis-trans isomerase,chloroplast	*Citrus aurantium*	19.41/9.63	19.26/5.88	116	100	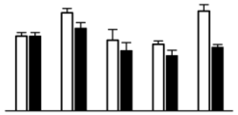
**Redox**								
b22	Gi|270230992	Thioredoxin m-type 4	*Vitis vinifera*	20.33/ 9.37	12.53/6.18	142	100	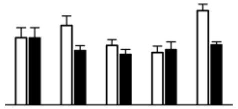
a9	Gi|42477789	Ankyrin repeat –containing protein 2	*Citrus sinensis*	16.29/5.27	49.80/4.34	377	100	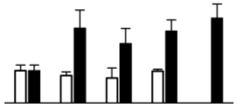

c7	Gi|209934640	Peroxiredoxin type 2, putative	*Citrus unshiu*	32.81/9.67	13.32/4.64	119	100	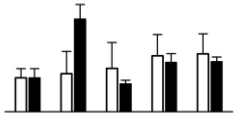
**Phtosynthesis**								
c8	Gi|57930837	PS II oxygen-evolving complex 1	*Citrus clementina*	31.72/5.22	32.58/5.22	215	100	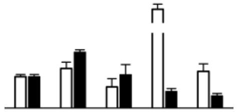
a10	Gi|225440438	Ribulose-1,5-bisphosphate carboxylase	*Vitis vinifera*	48.17/6.40	56.60/4.83	112	100	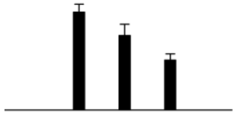
a15	Gi|115334979	Ribulose-1,5-bisphosphate carboxylase	*Acer rubrum*	47.95/7.57	56.17/5.01	375	100	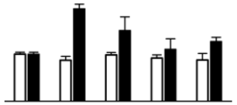
**Others**								
e4	Gi|55932194	In2-1 protein, putative	*Citrus sinensis*	32.43/8.24	29.61/5.17	120	100	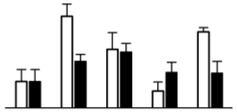
e10	Gi|188284839	Rhodanese-like domain-containing protein	*Citrus latifolia*	31.68/9.64	54.98/5.05	121	100	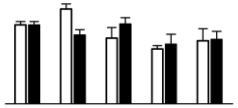
c9	Gi|116057804	Myosin class II heavy chain (ISS)	*Ostreococcus tauri*	468.49 /6.17	14.87/6.52	85	99.79	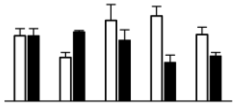
